# Association of socioeconomic and demographic determinants with clinical outcomes in Iraqi patients with diabetes: A cross-sectional study

**DOI:** 10.1371/journal.pgph.0005475

**Published:** 2025-11-21

**Authors:** Samer Imad Mohammed, Ahmad kadhim Al-Jalehawi, Mohammed Yawuz Jamal, Shahbaa Mohammed Ati

**Affiliations:** 1 Department of Clinical Pharmacy, College of Pharmacy, University of Baghdad, Baghdad, Iraq; 2 Clinical Pharmacy, University of Alkafeel, Iraq; 3 Clinical Pharmacy Board. Department of Clinical Pharmacy, College of Pharmacy, University of Baghdad, Baghdad, Iraq; 4 Pharmacy science, Department of Pharmaceutics, College of Pharmacy, University of Baghdad, Baghdad, Iraq; PLOS: Public Library of Science, UNITED STATES OF AMERICA

## Abstract

Social determinants of health (SDH) profoundly influence diabetes outcomes; nevertheless, their impact on the Iraqi diabetic population remains under researched. The objectives of this study were To investigate the relationship between particular social determinants of health (SDH) variables namely food and housing insecurity, social support, income, and education and clinical outcomes, including HbA1c levels, medication adherence, and patient satisfaction among Iraqi diabetic patients. A cross-sectional study involving 212 diabetic patients in Iraq was conducted. Participants attending a healthcare facility in Iraq filled out validated questionnaires regarding social determinants of health, medication adherence, and satisfaction. HbA1c readings were extracted from medical records. Data were examined utilizing Spearman’s correlation. The average HbA1c was 7.4% ± 2.7. A majority of individuals had moderate housing insecurity (79.2%) and low food insecurity (75%). The principal discovery was that no social determinants of health variables exhibited a significant connection with HbA1c levels. Patient satisfaction exhibited a positive correlation with social support (p < 0.001) and higher income (p = 0.023), while demonstrating a negative correlation with housing insecurity (p = 0.040). Social support was the sole factor substantially correlated with improved medication adherence (p = 0.003). In conclusion, SDH were not directly associated with diabetes control but significantly influenced patient-reported experiences. Social assistance and money increased contentment, whereas housing insecurity diminished it. Social support was a significant factor in drug adherence. The results underscore the necessity of addressing psychosocial and economic issues to enhance the quality of diabetes care in Iraq.

## Introduction

Increases in health care costs, morbidity, and death due to diabetes are becoming more of a problem around the world. The predicted rise in the prevalence of diabetes is among the highest in the Middle East and North African (MENA) region [1]. It was reported that more than 10% of Iraqis are diabetic, and the number of cases is steadily rising [2,3].

Although traditional risk factors like high blood sugar, high blood pressure, and dyslipidemia account for a substantial portion of the risk of diabetic complications, there is mounting evidence that social determinants of health also play a role in health outcomes [4].

According to World health organization (WHO), the social determinants of health (SDH) are “the non-medical factors that influence health outcomes. They are the conditions in which people are born, grow, work, live, and age, and the wider set of forces and systems shaping the conditions of daily life.” These forces include economic and political systems, development agendas, and social norms and policies [5].

Mitigating the health effects of social determinants requires a multisectoral understanding of their influence on individual and population health. Clinicians, policymakers, and communities should pursue structural interventions that combine broad policy implementation with targeted local solutions. Such approaches can diminish health inequities while advancing population health equity [6].

Health equity is a lens that is gaining traction at home to address the 50–60% of health outcomes that are influenced by social and environmental factors; these disparities fall under this umbrella on a global level. These factors, which are known as SDH, show that diabetes is a social illness in addition to a metabolic one [7].

The significant impact of social determinants of health (SDOH) on diabetes outcomes - reflected in the disease’s growing prevalence, substantial economic burden, and disproportionate effects on vulnerable populations [8]. has made their investigation a priority for research. This builds upon the American Diabetes Association’s (ADA) foundational 2013 scientific statement examining socioecological factors in prediabetes and type 2 diabetes [9].

Research indicates that the integration of medical care with interventions that address these social risk factors can enhance health outcomes. There is a scarcity of information regarding the effective identification of interventions by grouping social hazards [10].

Despite noting the risk factors in Iraq in addition to the importance of the quality of life, profoundly impacts physical health, social relationships, and the environment [11], the effect of the SDH was not examined.

Consequently, the objective of this research is to examine the correlation between certain SDH variables and the clinical outcomes, satisfaction with care, and medication adherence of Iraqi diabetic patients. This study therefore aims to fill this critical evidence gap by providing one of the first comprehensive assessments of how key SDH factors including education, income, food and housing insecurity, and social support influence glycemic control, treatment adherence, and patient satisfaction within the unique Iraqi healthcare context.

## Materials and methods

### Study design

The study employed a cross-sectional approach, utilizing specific questionnaires to assess various social determinants of health (SDH) factors, including food and housing instabilities, as well as social support, among a sample of diabetes mellitus (DM) patients. The sample size of 212 participants was determined based on feasibility and patient availability during the study period. Data collection was conducted over a nine-month period, from March 15 to December 25, 2024, which was the timeframe required to enroll the target sample from the patient population attending the clinic.

### Participants

Convenient sample of patients with DM who meet the following inclusion criteria: Adult patients who have been diagnosed with type 1 or type 2 diabetes and are currently receiving treatment at a healthcare facility. The study does not include any patient under the age of 18 or any patient who has not recently received HB1Ac. The exploratory nature of the study and practical constraints in patient recruitment necessitated the use of a convenience sampling method. This method effectively generated preliminary data on these novel relationships; however, we recognize its potential for selection bias and the resulting limitations on the generalizability of the findings, which are addressed in the limitations section.

### Ethics statement

The research proposal received ethical approval from the Central Scientific Committee at the University of Baghdad College of Pharmacy with an approval number(REC-23354–2023). Verbal informed consent was formally authorized by the committee. The researcher documented the process of obtaining verbal consent from each participant on a participant document, following a thorough description of the study. The survey was conducted anonymously, and participation in the study was voluntary.

### Data collection

In order to accumulate comprehensive data regarding participants’ sociodemographic attributes and SDH, this investigation implemented a self-administered questionnaire. The questionnaire was divided into two primary domains:[1] demographic variables, which included age, gender, educational attainment, and income level; and [2] validated SDH measures, which evaluated critical factors such as food insecurity, housing stability, and social support networks. Clinical data on glycemic control (HbA1c) were extracted from medical records to assess diabetes-related outcomes. Self-reported measures were used to capture patient satisfaction with diabetes care and medication adherence patterns.

### Questionnaires

1- Food Insecurity: The USDA 6-Item Short Form Food Security Survey Module, a validated instrument that evaluates household food access over the past 12 months, was employed to evaluate food insecurity. The six inquiries on this scale assess food-related hardships, including reduced intake, meal skipping, and hunger as a result of financial constraints.

Scoring: Each response is assigned a value: Always = 5, Often = 4, Sometimes = 3, Rarely = 2, Never = 1. Then, the scores of each question were added to determine the total score. Higher scores suggest a greater degree of food insecurity. The final results indicated low food insecurity if the sum ranged between 10 and 15. Values ranging from 16 to 20 indicate moderate food insecurity, whereas values between 21 and 30 signify high food insecurity [12].

2- Housing Stability Assessment

The Housing Stability Assessment questionnaire is a validated tool that assesses various aspects of housing insecurity, such as affordability, displacement history, and living conditions. This tool utilizes established surveys, including the National Health Interview Survey (CDC, 2023) and the Survey of Income and Program Participation (U.S. Census Bureau, 2023), to evaluate critical indicators such as rent or mortgage burden in relation to income, prior experiences with eviction or foreclosure, instances of homelessness, and housing adequacy, which encompasses factors like overcrowding and access to utilities.

Responses are obtained through a combination of 5-point Likert scales to assess perceived instability and binary (yes/no) items for specific events. Participants are classified into three categories based on total scores: stable (0–5), moderately unstable [6–10], and severely unstable [11]+. The questionnaire exhibits strong reliability (test-retest r = 0.89) and is extensively utilized in health research to investigate the relationships between housing instability and clinical outcomes. The standardized design facilitates cross-population comparisons and is adaptable to specific research contexts, including studies on chronic disease management where housing security may affect treatment adherence.

The questionnaire utilizes a dual scoring system to thoroughly assess housing instability. Initially, binary items (Yes/No) evaluate specific risk factors: each affirmative response to eviction history (Q1), foreclosure (Q2), homelessness/shelter use (Q3), or forced relocation (Q4) adds 1 point to the overall score. These dichotomous items represent objective, high-impact events that pose direct threats to housing security.

Secondly, Likert-scale items provide a quantifiable measure of subjective experiences. Responses to affordability stress (Q5) range from “Always” (4 points) to “Never” (0), indicating that higher scores correspond to increased financial strain. In contrast, neighborhood safety (Q6) employs reverse scoring, with “Always” assigned a value of 0 and “Never” a value of 4, indicating that lower scores reflect increased environmental risks. This method guarantees uniform interpretation, where elevated values consistently indicate greater instability for all items.

Respondents are classified into three tiers based on total scores ranging from 0 to 12. The initial range of 0–4 signifies low instability, characterized by minimal risk factors, serving as the reference group. A titer of 5–8 indicates moderate instability, necessitating monitoring due to multiple stressors. A titer of 9 or higher is considered indicative of high instability, which has been associated with adverse health outcomes in previous studies [13,14].

3- The Social Support Networks Assessment

This questionnaire is a validated tool designed to evaluate multiple dimensions of social support, including emotional support (e.g., having someone to confide in), instrumental support (e.g., practical help with daily needs), trust, companionship, and community belonging.

The Social Support Networks Assessment consists of six Likert-scale questions, where responses are scored from 1 (Never) to 5 (Always). The total score is calculated by summing all responses, with higher scores (range: 0–30) indicating stronger social support. Scores are interpreted as: 25–30 (High support), 15–24 (Moderate support), and 0–14 (Low support), providing a quick, reliable measure of an individual’s perceived social support strength [15,16].

### Diabetes-specific measures

Glycemic control was evaluated through the latest HbA1c level documented in medical records, offering an objective assessment of long-term blood glucose regulation. Medication adherence was assessed via self-report, where patients indicated the frequency of their adherence to prescribed diabetes medications using a 5-point Likert scale (Always, Often, Sometimes, Rarely, Never). Patient satisfaction was assessed by requesting participants to evaluate their overall satisfaction with diabetes care using a 5-point scale (Very satisfied, Satisfied, Neutral, Dissatisfied, very dissatisfied). The standardized measures facilitated a uniform evaluation of the clinical, behavioral, and experiential dimensions of diabetes management.

### Statistical analysis

Statistical analysis was conducted using version 26 of the Statistical Package for the Social Sciences (SPSS) software. Continuous variables were expressed as mean ± standard deviation (SD). The presentation of categorical variables included the depiction of percentages and frequencies. A probability value below 0.05 was considered statistically significant. The results’ normality was evaluated through a Shapiro-Wilk test, indicating that the data followed a normal distribution. Spearman’s Rank-Order Correlation was conducted to analyze the relationship between various SDH and patient HbA1c, adherence, and satisfaction. The independent variables were the SDH measures (food insecurity, housing insecurity, social support, income, education). The dependent variables were HbA1c levels, patient satisfaction, and medication adherence. No significant missing data was encountered in the collected questionnaires; complete case analysis was therefore performed.

## Results

The current study enrolled 212 diabetic patients, the majority of whom were female and had type 2 diabetes. The participants’ mean age was 46.67 ± 17 years. Table 1 illustrates additional demographic data.

**Table 1:** Demographic characteristics.

Main Category	Subcategory	Values
Age (year)	Mean ±SD	46.67 ± 17
Duration of DM (year)	Mean ±SD	10.767 ± 9.4
Sex	Male N (%).	103 (48.6)
	Female N (%).	109(51.4)
DM type	DM type 1 N (%).	75(35.4)
	DM type 2 N (%).	137 (64.6)
Education	Illiterate N (%).	7 (3.3)
	Elementary N (%).	22 (10.4)
	High school N (%).	51(24.1)
	Bachelor N (%).	102(48.1)
	Postgraduate studies N (%).	30 (14.1)
Income (ID) (Iraqi Dinar)	Less than 500K N (%).	77 (36.3)
	501-1M N (%).	16 (7.5)
	1.1-1.5M N (%).	104 (49.1)
	More than 1.5 N (%).	15 (7.1)
Residency	Urban N (%).	10 (4.7)
	City N (%).	202 (95.3)

DM: diabetes mellites. K: thousand. M: million.

Table 2 presents the primary clinical characteristics of the participants. The majority of the samples examined did not monitor their blood glucose levels on a daily basis. More than one-third of patients exhibited elevated fasting and random blood sugar levels, despite reporting good adherence and satisfaction.

**Table 2:** Clinical Characteristics.

Main Category	Subcategory	Values
Test Blood Sugar Daily	Yes	86 (40.6)
	No	126 (59.4)
Last fasting blood sugar test (mg/dl)	less than 100	39 (18.4)
	101-125	60 (28.3)
	126-140	41(19.3)
	More than 141	42 (19.8)
Last random blood sugar test(mg/dl)	less than 140	33 (15.6)
	141-160	58 (27.4)
	161-180	30 (14.2)
	more than 180	91(42.9)
Last Hb A1C	Mean± SD	7.4 ± 2.7
	less than 6.5	35 (16.5)
	6.6-7.5	70 (33)
	7.6-8.5	56 (26.4)
	8.6-9.5	32 (15.1)
	more than 9.5	19 (9)
Adherence (how often the patient takes the prescribed diabetes medications as directed)	Always	134 (63.2)
	Often	59 (27.8)
	Sometimes	19 (9)
	Rarely	0
	Never	0
Associated cardiovascular diseases	Yes	72 (34)
	No	140(66)
Patient satisfaction	Very dissatisfied	21 (10)
	Dissatisfied	8 (3.8)
	Neutral	46 (21.8)
	Satisfied	55 (26.1)
	Very satisfied	82 (38.4)

As shown in Table 3, participants exhibited variable food insecurity, with a mean value of 12.85 ± 3.94, indicating a low level of insecurity. The study found that the majority of the sample exhibited low levels of insecurity, scoring below 15, while fewer than 10% demonstrated high levels of insecurity, scoring above 21.

**Table 3:** Distribution of patients by food insecurity status.

Category	Value
Mean of values (Mean± SD)	12.85 ± 3.94
Patient with Low food insecurity No. (%)	159 (75)
Patient with Moderate food insecurity No. (%)	33 (15.6)
Patient with High food insecurity No. (%)	20 (9.4)

As seen in Table 4, the findings reveal that the average housing insecurity score was 5.71 ± 2, signifying a moderate level of insecurity, with 79.2% of the sample exhibiting moderate insecurity levels.

**Table 4:** Distribution of patients by housing insecurity Status.

Category	Value
Mean of values (Mean ±SD)	5.71 ± 2
Patient with Low housing insecurity No. (%)	30(14.2)
Patient with Moderate housing insecurity No. (%)	168(79.2)
Patient with High housing insecurity No. (%)	14 (6.6)

Table 5 indicates that most diabetes mellitus patients receive a modest degree of social assistance.

**Table 5:** Distribution of patients by social support Status.

Category	Value
Mean of values (Mean ±SD)	18.834 ± 3.21
Patient with low social support	16(7.5)
Patient with moderate social support	185 (87.3)
Patient with high social support	11 (5.2)

The analysis of the association between SDH and HbA1c levels reveals no significant correlation between the SDH variables and HbA1c.\. Neither patient income nor education level was correlated with HbA1c levels.. Patients’ satisfaction was adversely impacted by levels of housing insecurity; as insecurity increased, satisfaction decreased significantly. The findings indicated a significant positive correlation between patient satisfaction and both social support and higher income.. Medication adherence to medication regimens showed a significant positive correlation exclusively with social support. In contrast, food insecurity and housing insecurity did not present a statistically significant relationship with medication adherence, although both factors were noted to negatively impact adherence levels (Table 6).

**Table 6:** Correlation between SDH and clinical variables.

variable	SDH and HbA1c		SDH and patient satisfaction.		SDH and Medication adherence	
	Correlation Coefficient	P. value	Correlation Coefficient	P. value	Correlation Coefficient	P. value
Food insecurity	0.026	0.712	-0.080	0.249	-.045	0.519
Housing insecurity	-0.018	0.799	-0.136	0.040*	-.019	0.780
Social support	-0.045	0.514	0.429	<0.001*	0.31	0.003*
Income	0.023	0.735	0.157	0.023*	0.045	0.517
Education	-0.054	0.437	0.107	0.122	0.044	0.523

Spearman’s Rank-Order Correlation. *: significant correlation

## Discussion

### Summary of key findings

This research identified a clear pattern in the association between Social Determinants of Health (SDH) and diabetes outcomes within an Iraqi population. No social determinants of health variables, including education, income, food insecurity, housing insecurity, and social support, exhibited a significant correlation with glycemic control as indicated by HbA1c levels. Psychosocial and economic factors exhibited a strong association with patient-reported experiences. Higher social support and income were significantly correlated with increased patient satisfaction, whereas greater housing insecurity was associated with decreased satisfaction. Additionally, social support was identified as the only factor significantly linked to enhanced medication adherence. These findings highlight that although social determinants of health may not directly affect biological markers in this context, they are essential for patient-centered outcomes such as satisfaction and adherence.

### Comparison with existing literature and interpretation

The present investigation demonstrated that the majority of diabetic patients examined do not diligently monitor their blood glucose levels on a daily basis, which has a substantial effect on their disease management. Research suggests that a variety of factors, such as emotional barriers, financial constraints, and a lack of education, contribute to this non-adherence [17].

According to a study conducted by Patrick et al. (1994), 52% of patients did not participate in any type of home glucose monitoring. A non-adherence rate for self-monitoring of 45.94% was reported in another study, with Type 2 diabetes exhibiting a higher rate of 47.15% [18].

Financial concerns were identified as a major barrier, with 80% of patients citing the cost of testing strips as prohibitive [18].

Additionally, emotional and educational factors also played a role as presented by Tientcheu et al. study which showed that 60% of patients were unaware of the importance of self-monitoring [17].

Although the majority of diabetic patients in the current study report that they adhere to their treatment plans and are satisfied with their care, over one-third continue to experience elevated fasting and random blood sugar levels. This perplexing discrepancy between effort and outcomes is the consequence of numerous underlying factors that influence blood sugar regulation. Research indicates that patients may believe they are adhering to their medication regimen persistently; however, real-world compliance frequently fails to meet expectations. For instance, a study conducted by Despras et al. (2022) demonstrated that two-thirds of patients failed to adhere to their insulin regimen, which led to critically elevated blood sugar levels [19].

However, diabetes management necessitates more than just medication administration; it necessitates substantial lifestyle modifications. Regrettably, only approximately 22% of patients adhere to their prescribed physical activity, as evidenced by a study conducted by Marinho et al. (2018) [20].

This emphasizes the necessity of a more comprehensive approach to diabetes care that assists patients in making enduring, healthy daily decisions, in addition to ensuring that they take their medications.

Research has established a clear connection between food insecurity and poor glycemic control, with multiple studies indicating that individuals experiencing food insecurity often show higher hemoglobin A1c (HbA1c) levels and deteriorating diabetes-related health outcomes [21,22].

However, the current study’s findings indicate no association between the level of food insecurity and HbA1c, with participants reporting overall low levels of food insecurity, reflected in a mean score of 12.85 (±3.94). This indicates that, although food insecurity is a significant factor affecting glycemic management in larger populations, the current sample was mainly defined by mild to moderate insecurity and only a minor percentage (less than 10%) scored above 21, signifying severe food insecurity. Recent evidence underscores the negative effects of food insecurity on glycemic control among various age groups and populations. A recent study on U.S. adolescents indicated that food insecurity was linked to slightly increased HbA1c levels, implying its potential as a risk factor for early metabolic dysregulation in this demographic [23].

Research among adults with type 2 diabetes consistently showed a correlation between food insecurity and suboptimal glycemic management, indicated by elevated HbA1c levels [22,24].

The findings highlight that food insecurity, in both adolescence and adulthood, can adversely affect diabetes-related outcomes by restricting access to nutritious foods and hindering effective self-care practices. The varying mechanisms across age groups highlight the consistent link between food insecurity and inadequate glycemic control, underscoring the necessity for targeted interventions that prioritize nutritional stability in diabetes management.

Evidence increasingly indicates that housing insecurity significantly deteriorates diabetes outcomes by introducing various obstacles to effective disease management [25].

Studies show that this population encounters significant difficulties in achieving glycemic control, obtaining regular medical care, and following self-care practices, which results in heightened acute healthcare utilization [26,27].

Research indicates that housing-insecure individuals have increased rates of emergency department visits and hospitalizations due to diabetes-related crises, as well as a higher incidence of severe complications such as diabetic ketoacidosis. Intervention studies indicate that rental assistance programs and supportive housing initiatives yield significant improvements in diabetes management, evidenced by increased hemoglobin A1c monitoring and enhanced engagement with diabetes care services [28,29].

The findings highlight housing stability as a significant social determinant of health for populations with diabetes. The results of the current study indicate that 79.2% of diabetes patients surveyed experienced moderate housing insecurity, with a mean score of 5.71 ± 2.

Although the current study analysis of the association between housing insecurity and HbA1c or Medication adherence levels reveals no significant correlation. However, levels of housing insecurity adversely impacted patients’ satisfaction; as insecurity increased, satisfaction significantly decreased.

While the current study found no statistically significant association between housing insecurity and either HbA1c levels or medication adherence metrics, it revealed important psychosocial impacts on diabetes management. The analysis demonstrated a clear inverse relationship between housing insecurity and patient satisfaction, with satisfaction levels declining significantly as housing instability increased. This finding suggests that while housing insecurity may not directly manifest in conventional biomarkers of glycemic control, it exerts substantial negative effects on patients’ perceived quality of care and overall healthcare experience. The moderate housing insecurity observed in 79.2% of participants (mean score 5.71 ± 2) thus represents an important psychosocial determinant of diabetes care quality, even in the absence of direct metabolic correlates. This distinction underscores the need for multidimensional assessment of diabetes outcomes that incorporates both clinical indicators and patient-reported experiences.

Numerous studies have demonstrated that housing insecurity adversely affects diabetes management outcomes, including HbA1c levels, medication adherence, and patient satisfaction, influenced by factors such as financial strain, stress, food insecurity, and limited access to healthcare [28,30,31].

Housing insecurity is associated with greater diabetes distress, depression, and anxiety, which negatively impact diabetes management outcomes as addressed by Hundt et al study [32].

Moreover, housing instability can diminish self-efficacy in diabetes, an essential component for effective disease management. Reduced self-efficacy correlates with adverse health outcomes and may diminish patient satisfaction [33].

Social support is a crucial and variable factor in diabetes management, significantly affecting psychological well-being and adherence to treatment. Our findings indicate that the majority of diabetes patients receive limited social assistance, aligning with prior research. A study conducted at Indramayu Hospital revealed a nearly equal distribution of social support among type 2 diabetes patients, with 54.72% experiencing low social support and 45.28% indicating high support, highlighting significant variability in support networks [34].

Regional differences are notable, as indicated by data from Central Karnataka, which reveals higher support levels (58% high support compared to 42% moderate) [35].

Social support positively correlates with patient satisfaction and medication adherence among participants in the current study. This relationship is essential, as medication adherence significantly influences effective diabetes management and the prevention of complications. Numerous studies have examined the influence of social support on medication adherence and patient satisfaction, underscoring its importance in diabetes management. A study in China demonstrated that social support significantly affects medication adherence in patients with type 2 diabetes mellitus (T2DM). The study indicated that the use of support and particular subscales of social support significantly influenced adherence, implying that social support ought to be a fundamental component in interventions designed to enhance diabetes management [36].

Research in Ghana indicated a positive correlation between social support and self-management behaviors, such as medication adherence, in patients with Type 2 Diabetes Mellitus (T2DM) [37].

Improved social support is associated with enhanced psychological outcomes and increased patient satisfaction. According to the study by Presley et al., patients who express greater satisfaction with social support exhibit reduced levels of diabetes distress [38].

### Study Strengths, Implications, and Future Directions

This study’s primary strength lies in its innovative emphasis on social determinants of health within the inadequately studied Iraqi diabetic population, utilizing validated instruments for a comprehensive evaluation. The findings suggest that clinical practice in Iraq should incorporate routine psychosocial support and screening for housing instability to improve patient-centered care. This highlights the necessity for policies that enhance social safety nets in public health. Future research should utilize longitudinal designs with probability sampling across various Iraqi centers to establish causality and enhance generalizability, while further investigating the intricate relationships between social determinants of health and clinical outcomes.

### Limitations

The reliance on a convenience sample from a single center may restrict the applicability of our findings to the wider Iraqi diabetic population. The cross-sectional design limits the ability to establish causality. Future research should utilize stratified random sampling across various centers to improve the representativeness.

## Conclusion

There is no significant correlation between HbA1c levels and individual SDH variables (education, income, food/housing insecurity, and social support). Nevertheless, patient satisfaction was inversely correlated with housing insecurity, whereas social support and income had a positive impact on satisfaction. Social support was the sole factor that significantly influenced medication adherence. Despite the fact that food and housing insecurity had a detrimental effect on adherence, these associations were not statistically significant. The findings highlight the importance of social support and housing security in relation to patient satisfaction and medication adherence, while also revealing a complex relationship between social determinants of health and glycemic control in this population. The findings indicate that public health initiatives and clinical care models for diabetes in Iraq must extend beyond a solely biomedical approach. To enhance patient experiences and adherence, programs ought to incorporate psychosocial support systems, including peer support groups and community health worker interventions. Policymakers and clinicians should incorporate screening for housing instability into standard diabetes care and establish collaborations with social services to address this critical factor influencing patient satisfaction.

### Declaration of generative AI and AI-assisted technologies in the writing process

During the preparation of this work, the authors used [Quill Bot/ SERVICE] to [rephrase]. After using this tool/service, the authors reviewed and edited the content as needed and took full responsibility for the content of the publication.

## Supporting information

S1 Data Dataset. (PDF)
