# Supplementary material for: Association of socioeconomic and demographic determinants with clinical outcomes in Iraqi patients with diabetes: A cross-sectional study
Source: PLOS Glob Public Health. 2025 Nov 21;5(11):e0005475. doi: 10.1371/journal.pgph.0005475 (PMC12637932; doi:10.1371/journal.pgph.0005475)
Supplement: S1 Data — (PDF) [file pgph.0005475.s001.pdf]

No.	Age (years)	Gender	DM type	Education	income
1	55	Male	DM Type II	Bachelor	1.001M - 1.5 M
2	54	Female	DM Type II	Elementary	<500K
3	56	Male	DM Type I	High School	501K-1 M
4	55	Male	DM Type II	High School	501K-1 M
5	55	Female	DM Type II	Elementary	501K-1 M
6	29	Male	DM Type II	Bachelor	1.001M - 1.5 M
7	38	Female	DM Type I	High School	<500K
8	46	Male	DM Type I	Elementary	501K-1 M
9	65	Male	DM Type II	diploma	501K-1 M
10	54	Female	DM Type I	High School	<500K
11	33	Male	DM Type II	Bachelor	> 1.5 M
12	50	Female	DM Type II	Bachelor	1.001M - 1.5 M
13	60	Female	DM Type II	Illiterate	<500K
14	62	Male	DM Type I	Bachelor	501K-1 M
15	19	Female	DM Type I	Bachelor	501K-1 M
16	65	Female	DM Type II	Bachelor	<500K
17	45	Male	DM Type I	High School	<500K
18	33	Male	DM Type II	Bachelor	<500K
19	40	Female	DM Type II	High School	1.001M - 1.5 M
20	47	Male	DM Type I	Elementary	501K-1 M
21	37	Female	DM Type I	High School	<500K
22	36	Male	DM Type I	High School	501K-1 M
23	55	Male	DM Type II	Bachelor	1.001M - 1.5 M
24	53	Female	DM Type I	High School	<500K
25	31	Male	DM Type I	Bachelor	> 1.5 M
26	50	Female	DM Type I	Bachelor	> 1.5 M
27	61	Female	DM Type II	High School	1.001M - 1.5 M
28	52	Male	DM Type II	High School	501K-1 M
29	51	Female	DM Type II	High School	1.001M - 1.5 M
30	30	Male	DM Type I	Bachelor	1.001M - 1.5 M
31	36	Female	DM Type I	Bachelor	501K-1 M
32	45	Male	DM Type I	Bachelor	501K-1 M
33	57	Male	DM Type I	High School	1.001M - 1.5 M
34	54	Male	DM Type II	PhD	> 1.5 M
35	52	Male	DM Type II	diploma	1.001M - 1.5 M
36	22	Male	DM Type II	High school	<500K
37	50	Male	DM Type II	High school	<500K
38	55	Male	DM Type II	PhD	1.001M - 1.5 M
39	54	Male	DM Type II	PhD	1.001M - 1.5 M
40	22	Male	DM Type I	Bachelor	1.001M - 1.5 M
41	50	Male	DM Type II	Bachelor	<500K
42	65	Female	DM Type I	Elementary	<500K
43	27	Female	DM Type II	Bachelor	1.001M - 1.5 M
44	55	Male	DM Type II	Master	<500K
45	40	Male	DM Type II	Master	1.001M - 1.5 M
46	57	Female	DM Type II	Bachelor	<500K
47	21	Female	DM Type I	Bachelor	<500K
48	21	Female	DM Type I	Bachelor	<500K

49	22	Female	DM Type II	Bachelor	1.001M - 1.5 M
50	64	Male	DM Type II	Bachelor	1.001M - 1.5 M
51	56	Female	DM Type II	High school	<500K
52	56	Female	DM Type II	High school	<500K
53	51	Male	DM Type I	Bachelor	1.001M - 1.5 M
54	22	Male	DM Type I	Bachelor	<500K
55	50	Male	DM Type II	Bachelor	<500K
56	24	Female	DM Type I	Bachelor	1.001M - 1.5 M
57	30	Female	DM Type I	Bachelor	1.001M - 1.5 M
58	71	Male	DM Type II		<500K
59	54	Female	DM Type II	Bachelor	1.001M - 1.5 M
60	47	Female	DM Type II	Bachelor	1.001M - 1.5 M
61	21	Female	DM Type II	Bachelor	> 1.5 M
62	22	Female	DM Type II		> 1.5 M
63	63	Female	DM Type II	Bachelor	1.001M - 1.5 M
64	75	Male	DM Type II	Bachelor	1.001M - 1.5 M
65	60	Male	DM Type II	Elementary	<500K
66	60	Male	DM Type II	Bachelor	1.001M - 1.5 M
67	46	Female	DM Type II	Bachelor	501K-1 M
68	58	Female	DM Type II	High school	1.001M - 1.5 M
69	63	Male	DM Type II	diploma	<500K
70	40	Female	DM Type II	High School	<500K
71	49	Female	DM Type II	High School	<500K
72	22	Female	DM Type I	Bachelor	<500K
73	59	Male	DM Type II	High School	1.001M - 1.5 M
74	56	Male	DM Type II	High School	<500K
75	56	Male	DM Type II	High School	<500K
76	32	Female	DM Type II	Bachelor	<500K
77	59	Female	DM Type II	Bachelor	1.001M - 1.5 M
78	56	Male	DM Type II	High School	<500K
79	80	Female	DM Type II	Elementary	<500K
80	65	Female	DM Type II	diploma	501K-1 M
81					<500K
82	53	Male	DM Type II	High School	1.001M - 1.5 M
83	48	Male	DM Type II	Bachelor	1.001M - 1.5 M
84	56	Male	DM Type II	High School	> 1.5 M
85	46	Female	DM Type I	High school	1.001M - 1.5 M
86	46	Male	DM Type I	Bachelor	> 1.5 M
87	58	Female	DM Type II	Elementary	<500K
88	70	Female	DM Type I	High school	1.001M - 1.5 M
89	65	Female	DM Type II	High School	<500K
90	75	Male	DM Type II	Bachelor	<500K
91	18	Male	DM Type II	High school	<500K
92	50	Male	DM Type II	High School	1.001M - 1.5 M
93	62	Female	DM Type II	diploma	1.001M - 1.5 M
94	56	Female	DM Type II	Bachelor	1.001M - 1.5 M
95	25	Male	DM Type I	Bachelor	<500K
96	20	Female	DM Type I	Bachelor	1.001M - 1.5 M
97	20	Female	DM Type I	Bachelor	1.001M - 1.5 M

98	21	Female	DM Type I	Bachelor	1.001M - 1.5 M
99	20	Female	DM Type I	Bachelor	1.001M - 1.5 M
100	20	Female	DM Type I	Bachelor	1.001M - 1.5 M
101	53	Male	DM Type II	Bachelor	<500K
102		Female	DM Type II	Elementary	<500K
103	36	Male	DM Type II	Bachelor	> 1.5 M
104	48	Female	DM Type II	High School	> 1.5 M
105	30		DM Type I	Bachelor	1.001M - 1.5 M
106	50	Female	DM Type II	Bachelor	1.001M - 1.5 M
107	30	Male	DM Type I	Bachelor	1.001M - 1.5 M
108	22	Female	DM Type II	Bachelor	<500K
109	20	Female	DM Type I	Bachelor	1.001M - 1.5 M
110	20	Female	DM Type I	Bachelor	1.001M - 1.5 M
111	62	Male	DM Type II	Bachelor	1.001M - 1.5 M
112	20	Female	DM Type I	Bachelor	1.001M - 1.5 M
113	53	Male	DM Type II	Bachelor	1.001M - 1.5 M
114	70	Male	DM Type II	diploma	1.001M - 1.5 M
115	60	Female	DM Type II	High School	<500K
116	72	Female	DM Type II	High School	1.001M - 1.5 M
117	79	Male	DM Type II	Bachelor	501K-1 M
118	67	Female	DM Type I	Elementary	<500K
119	45	Male	DM Type II	Bachelor	1.001M - 1.5 M
120	67	Female	DM Type I	Elementary	<500K
121	67	Female	DM Type I	Elementary	<500K
122	55	Female	DM Type II	diploma	1.001M - 1.5 M
123	74	Female	DM Type II	Illiterate	<500K
124	70	Male	DM Type I	diploma	1.001M - 1.5 M
125	22	Female	DM Type I	Bachelor	1.001M - 1.5 M
126	64	Female	DM Type II	diploma	1.001M - 1.5 M
127	22	Female	DM Type I	Bachelor	1.001M - 1.5 M
128	60	Male	DM Type II	High School	<500K
129	55	Male	DM Type II	Bachelor	1.001M - 1.5 M
130	55	Female		Illiterate	<500K
131	23	Male	DM Type I	Bachelor	1.001M - 1.5 M
132	21	Female	DM Type I	Bachelor	> 1.5 M
133	53	Female	DM Type II	Bachelor	1.001M - 1.5 M
134	21	Female	DM Type I	Bachelor	1.001M - 1.5 M
135	21	Female	DM Type I	Bachelor	1.001M - 1.5 M
136	21	Female	DM Type I	Bachelor	1.001M - 1.5 M
137	60	Female	DM Type II	Illiterate	1.001M - 1.5 M
138	70	Male	DM Type II	Bachelor	501K-1 M
139	59	Male	DM Type II	Bachelor	> 1.5 M
140	58	Male	DM Type II	Bachelor	1.001M - 1.5 M
141	60	Female	DM Type II	Elementary	<500K
142	53	Male	DM Type II	Master	1.001M - 1.5 M
143	59	Female	DM Type II	High School	<500K
144	23	Female		Bachelor	<500K
145	23	Female	DM Type I	Bachelor	<500K
146	21	Female	DM Type I	Bachelor	> 1.5 M
147		Female	DM Type II	Elementary	1.001M - 1.5 M
148	21	Female	DM Type II	Bachelor	<500K
149	44	Female	DM Type II	Elementary	<500K
150	21	Male			1.001M - 1.5 M
151	22	Male	DM Type I	Bachelor	1.001M - 1.5 M
152	70	Male	DM Type II	diploma	<500K
153	23	Female	DM Type II	Bachelor	1.001M - 1.5 M
154	56	Female	DM Type II	High School	<500K
155	14	Male	DM Type I	High School	1.001M - 1.5 M

156	54	Male	DM Type II	Bachelor	1.001M - 1.5 M
157	49	Male	DM Type II	Bachelor	1.001M - 1.5 M
158	21	Male	DM Type II	Bachelor	<500K
159	24	Female	DM Type II	Bachelor	<500K
160	80	Female	DM Type II		<500K
161	22	Male	DM Type I	Bachelor	1.001M - 1.5 M
162	45	Male	DM Type II	High School	1.001M - 1.5 M
163	65	Female	DM Type II	High School	1.001M - 1.5 M
164	55	Male	DM Type I	Bachelor	1.001M - 1.5 M
165	38	Male	DM Type I	Elementary	<500K
166	48	Male	DM Type I	High School	1.001M - 1.5 M
167	22	Female	DM Type I	Bachelor	<500K
168	71	Male	DM Type II	Bachelor	1.001M - 1.5 M
169	55	Male	DM Type II	Bachelor	1.001M - 1.5 M
170	53	Female		diploma	<500K
171	46	Female	DM Type II	High school	1.001M - 1.5 M
172	24	Male	DM Type I	Bachelor	<500K
173	45	Male	DM Type II	High School	1.001M - 1.5 M
174	55	Female	DM Type II		1.001M - 1.5 M
175	40	Male	DM Type II	Bachelor	1.001M - 1.5 M
176	21	Male	DM Type II	Bachelor	<500K
177	22	Female	DM Type I	Bachelor	<500K
178	23	Female	DM Type II	Bachelor	<500K
179	52	Male	DM Type II	High school	<500K
180	49	Female	DM Type II	Elementary	1.001M - 1.5 M
181	34	Female	DM Type I	Elementary	1.001M - 1.5 M
182	70	Female	DM Type I	Bachelor	1.001M - 1.5 M
183	60	Female	DM Type I	Illiterate	<500K
184	17	Male	DM Type I	High scool	<500K
185	61	Male	DM Type II	High scool	<500K
186	60	Female	DM Type II	Illiterate	<500K
187	55	Female	DM Type II	Elementary	<500K
188	55	Female	DM Type II	Elementary	<500K
189	70	Female	DM Type II	Diploma	1.001M - 1.5 M
190	42	Male	DM Type II	Diploma	1.001M - 1.5 M
191	63	Female	DM Type II	Bachelor	1.001M - 1.5 M
192	68	Male	DM Type II	High school	1.001M - 1.5 M
193	68	Male	DM Type II	Master	> 1.5 M
194	75	Male	DM Type I		1.001M - 1.5 M
195	31	Female	DM Type II	Bachelor	1.001M - 1.5 M
196	41	Male	DM Type II	Bachelor	1.001M - 1.5 M
197	28	Female	DM Type I	Bachelor	1.001M - 1.5 M
198	55	Male	DM Type II	High School	1.001M - 1.5 M
199	60	Male	DM Type II	Bachelor	1.001M - 1.5 M
200	76	Female	DM Type II	High scool	<500K
201	45	Female	DM Type II	elementary	<500K
202	56	Male	DM Type II	High School	<500K
203	56	Male	DM Type II	Bachelor	1.001M - 1.5 M
204	60	Female	DM Type II	High school	1.001M - 1.5 M
205	22	Male	DM Type II	Bachelor	<500K
206	65	Male	DM Type II	Diploma	1.001M - 1.5 M
207	58	Female	DM Type II	Illiterate	1.001M - 1.5 M
208	51	Female	DM Type I	Elementary	1.001M - 1.5 M
209	48	Male	DM Type II	diploma	1.001M - 1.5 M
210	25	Female	DM Type II	Bachelor	1.001M - 1.5 M
211	48	Male	DM Type II	Bachelor	> 1.5 M
212	58	Male	DM Type I	diploma	1.001M - 1.5 M

Residency	Test Blood Sugar Daily ?	No. Daily test	Last fasting test	Last Random test	Last HbA1C
City	NO	NO Daily test	101 - 125	<140	6.6-7.5
City	NO	NO Daily test	>180	>180	6.6-7.5
City	NO	NO Daily test	>180	>180	7.6-8.5
City	NO	NO Daily test	>180	>180	6.6-7.5
City	NO	NO Daily test	141 - 180	>180	>9.6
City	NO	NO Daily test	<100	<140	7.6-8.5
City	NO	NO Daily test	141 - 180	<140	7.6-8.5
City	NO	NO Daily test	>180	>180	
City	NO	NO Daily test	>180	<140	7.6-8.5
City	YES	Twice	>180	141 - 160	>9.6
City	NO	NO Daily test	141 - 180	<140	6.6-7.5
City	NO	NO Daily test	141 - 180	>180	7.6-8.5
City	YES	Once	<100	>180	<6.5
City	YES	Once	126 - 140	141 - 160	<6.5
Urban	NO	NO Daily test	<100	<140	8.6-9.5
City	YES	Twice	126 - 140	>180	<6.5
City	NO	NO Daily test	>180	>180	>9.6
City	NO	NO Daily test	141 - 180	<140	6.6-7.5
City	YES	Once	101 - 125	141 - 160	6.6-7.5
City	NO	NO Daily test	>180	>180	>9.6
City	NO	NO Daily test	141 - 180	<140	7.6-8.5
City	NO	NO Daily test	>180	>180	6.6-7.5
City	NO	NO Daily test	>180	<140	7.6-8.5
City	YES	Twice	>180	141 - 160	>9.6
City	NO	NO Daily test	141 - 180	<140	7.6-8.5
City	NO	NO Daily test	141 - 180	>180	8.6-9.5
City	NO	NO Daily test	<100	>180	6.6-7.5
City	NO	NO Daily test	>180	>180	<6.5
City	NO	NO Daily test	141 - 180	>180	>9.6
City	NO	NO Daily test	<100	<100	8.6-9.5
City	NO	NO Daily test	141 - 180	141 - 160	8.6-9.5
City	YES	Once	>180	>180	8.6-9.5
City	YES	Once	126 - 140	>180	6.6-7.5
City	YES	Twice	101 - 125	141 - 160	6.6-7.5
City	NO	NO Daily test	101 - 125	<140	6.6-7.5
City	YES	Once	126 - 140	141 - 160	6.6-7.5
City	YES	Once	101 - 125	161 - 180	8.6-9.5
City	NO	NO Daily test	126 - 140	161 - 180	7.6-8.5
City	NO	NO Daily test	101 - 125	>180	6.6-7.5
City	YES	Trice	<100	141 - 160	<6.5
City	NO	NO Daily test	>180	>180	7.6-8.5
Urban	YES	Trice	141 - 180	>180	6.6-7.5
City	YES	Once	126 - 140	141 - 160	7.6-8.5
City	NO	NO Daily test	101 - 125	141 - 160	6.6-7.5
City	NO	NO Daily test	101 - 125	161 - 180	<6.5
City	YES	Once	126 - 140	141 - 160	6.6-7.5
City	YES	Twice	101 - 125	141 - 160	6.6-7.5
City	NO	NO Daily test	<100	<140	<6.5

City	YES	NO Daily test	141 - 180	>180	<6.5
City	NO	NO Daily test	126 - 140	161 - 180	6.6-7.5
City	NO	NO Daily test	>180	>180	8.6-9.5
City	NO	NO Daily test	>180	>180	8.6-9.5
City	NO		126 - 140	161 - 180	6.6-7.5
City	YES	>3 times	141 - 180	>180	7.6-8.5
City	NO	NO Daily test	>180	>180	7.6-8.5
City	NO	NO Daily test	126 - 140	141 - 160	<6.5
Urban	NO	NO Daily test	126 - 140	161 - 180	8.6-9.5
City	NO	Once	<100	>180	8.6-9.5
City	YES	Once	141 - 180	>180	8.6-9.5
City	YES	Once	141 - 180	>180	7.6-8.5
City	YES	Once	101 - 125	>180	
City	YES	Once	<100	>180	8.6-9.5
City	NO	NO Daily test	141 - 180	>180	
City	YES	Once	<100	>180	7.6-8.5
Urban	NO	NO Daily test	141 - 180	>180	8.6-9.5
City	NO	NO Daily test	101 - 125	161 - 180	<6.5
City	YES	Trice	126 - 140	141 - 160	6.6-7.5
City	YES	Once	126 - 140	161 - 180	6.6-7.5
City	NO	NO Daily test	<100	<140	6.6-7.5
City	YES	Once	101 - 125	<140	6.6-7.5
City	NO	NO Daily test	<100	161 - 180	<6.5
City	YES	Once	126 - 140	<140	6.6-7.5
City	YES	>3 times	<100	141 - 160	<6.5
City	YES	Once	141 - 180	>180	6.6-7.5
City	YES	Twice	126 - 140	>180	6.6-7.5
City	NO	NO Daily test	>180	>180	>9.6
City	NO	NO Daily test	126 - 140	>180	7.6-8.5
City	YES	Once	126 - 140	>180	6.6-7.5
City	YES	Trice	<100	<140	<6.5
City	NO	NO Daily test	101 - 125	161 - 180	6.6-7.5
City	NO	NO Daily test	141 - 180	>180	7.6-8.5
City	YES	Once	126 - 140		7.6-8.5
City	NO	NO Daily test	126 - 140	161 - 180	8.6-9.5
City	YES	Once	126 - 140	141 - 160	6.6-7.5
City	YES	Trice	<100	141 - 160	7.6-8.5
City	YES	Twice	101 - 125	141 - 160	7.6-8.5
City	YES	Trice	101 - 125	161 - 180	7.6-8.5
City	YES	Twice	101 - 125	141 - 160	6.6-7.5
City	YES	NO Daily test	141 - 180	>180	6.6-7.5
City	YES	Once	126 - 140	141 - 160	6.6-7.5
City	NO	NO Daily test	126 - 140	161 - 180	7.6-8.5
City	YES	Twice	101 - 125	161 - 180	8.6-9.5
City	NO	Once	101 - 125	141 - 160	<6.5
City	YES	Twice	101 - 125	141 - 160	7.6-8.5
City	YES	Trice	<100	>180	<6.5
City	NO	Trice	101 - 125	141 - 160	8.6-9.5
City	NO	Trice	101 - 125	141 - 160	8.6-9.5

City	NO	Trice	101 - 125	141 - 160	8.6-9.5
City	NO	Trice	101 - 125	141 - 160	8.6-9.5
City	NO	Trice	101 - 125	141 - 160	8.6-9.5
City	NO	Once	126 - 140	141 - 160	7.6-8.5
City	YES	Once	101 - 125	141 - 160	6.6-7.5
City	NO	NO Daily test	141 - 180	>180	6.6-7.5
City	NO	NO Daily test	<100	<140	<6.5
Urban	YES	>3 times	141 - 180	>180	6.6-7.5
City	NO	NO Daily test	101 - 125	141 - 160	6.6-7.5
Urban	YES	>3 times	141 - 180	>180	6.6-7.5
City	NO	NO Daily test	141 - 180	>180	8.6-9.5
City	NO	Trice	101 - 125	141 - 160	8.6-9.5
City	NO	Trice	101 - 125	141 - 160	8.6-9.5
City	YES	Once	141 - 180	>180	6.6-7.5
City	NO	Trice	101 - 125	141 - 160	8.6-9.5
City	YES	Twice	126 - 140	161 - 180	6.6-7.5
Urban	YES	Once	126 - 140	>180	>9.6
City	YES	Once	101 - 125	>180	<6.5
City	NO	NO Daily test	141 - 180	>180	<6.5
City	NO	NO Daily test	126 - 140	141 - 160	7.6-8.5
City	YES	Once	141 - 180	>180	<6.5
City	YES	Twice	101 - 125	141 - 160	6.6-7.5
City	YES	Once	141 - 180	>180	<6.5
City	YES	Once	141 - 180	>180	<6.5
City	YES	Once	<100	141 - 160	8.6-9.5
City	YES	Once	101 - 125	141 - 160	<6.5
City	YES	Once	<100	<140	6.6-7.5
City	YES	Trice	<100	<140	6.6-7.5
City	NO	Once	141 - 180	>180	>9.6
City	YES	Trice	<100	<140	6.6-7.5
City	YES	>3 times	126 - 140	161 - 180	8.6-9.5
City	YES	Once	126 - 140	141 - 160	6.6-7.5
City	NO	NO Daily test	>180	>180	
City	YES	Twice	141 - 180	>180	7.6-8.5
City	NO	NO Daily test	126 - 140	141 - 160	<6.5
City	YES	NO Daily test	101 - 125	141 - 160	<6.5
City	NO	Trice	101 - 125	141 - 160	8.6-9.5
City	NO	Trice	101 - 125	141 - 160	8.6-9.5
City	NO	Trice	101 - 125	141 - 160	8.6-9.5
City	YES	>3 times	<100	>180	6.6-7.5
City	YES	Twice	126 - 140	>180	>9.6
City	NO	NO Daily test	101 - 125	>180	7.6-8.5
City	NO	NO Daily test	141 - 180	161 - 180	6.6-7.5
City	NO	NO Daily test	141 - 180	>180	7.6-8.5
City	NO	NO Daily test	126 - 140	161 - 180	6.6-7.5
City	NO	NO Daily test	>180	>180	7.6-8.5
City	NO	NO Daily test	<100	141 - 160	<6.5
City	NO	NO Daily test	<100	141 - 160	7.6-8.5
City	NO	NO Daily test	<100	141 - 160	<6.5
City	NO	NO Daily test	126 - 140	>180	7.6-8.5
City	NO	NO Daily test	<100	<140	<6.5
Urban	YES	Once	101 - 125	<140	6.6-7.5
City	NO	NO Daily test	<100	<140	<6.5
City	YES	Twice	101 - 125	161 - 180	6.6-7.5
City	YES	Once	126 - 140	161 - 180	7.6-8.5
City	NO	NO Daily test	>180	>180	7.6-8.5
City	NO	NO Daily test	126 - 140	>180	>9.6
City	YES	Twice	141 - 180	>180	6.6-7.5

City	NO	NO Daily test	141 - 180	>180	6.6-7.5
City	NO	NO Daily test	126 - 140	141 - 160	6.6-7.5
City	YES	Trice	<100	>180	7.6-8.5
City	NO	NO Daily test	<100	161 - 180	<6.5
City	YES	Once	>180	>180	
City	YES	Twice	126 - 140	161 - 180	6.6-7.5
City	NO	NO Daily test	141 - 180	>180	7.6-8.5
City	NO	NO Daily test	101 - 125	<140	6.6-7.5
City	NO	NO Daily test	141 - 180	141 - 160	8.6-9.5
City	NO	NO Daily test	141 - 180	161 - 180	7.6-8.5
City	NO	NO Daily test	101 - 125	141 - 160	6.6-7.5
City	YES	Once	<100	<140	6.6-7.5
City	NO	NO Daily test	126 - 140	161 - 180	7.6-8.5
City	NO	NO Daily test	126 - 140	161 - 180	7.6-8.5
City	NO	NO Daily test	<100	<140	<6.5
City	NO	NO Daily test	<100	<140	<6.5
City	YES	Trice	126 - 140	>180	7.6-8.5
City	YES	Trice	101 - 125	141 - 160	6.6-7.5
City	YES	Once	141 - 180	>180	7.6-8.5
City	YES	Once	101 - 125	141 - 160	7.6-8.5
City	NO	NO Daily test	101 - 125	<140	<6.5
City	YES	Once	126 - 140	141 - 160	7.6-8.5
City	YES	Once	<100	141 - 160	6.6-7.5
City	NO	NO Daily test	101 - 125	<140	<6.5
City	YES	Once	>180	>180	>9.6
City	NO	NO Daily test	<100	>180	7.6-8.5
City	NO	NO Daily test	101 - 125	>180	6.6-7.5
City	NO	NO Daily test	101 - 125	>180	7.6-8.5
City	NO	NO Daily test	101 - 125	>180	6.6-7.5
City	NO	NO Daily test	>180		
City	NO	NO Daily test	>180	>180	>9.6
City	NO	NO Daily test	101 - 125	>180	6.6-7.5
City	NO	NO Daily test	>180	>180	>9.6
City	YES	Trice	101 - 125	>180	6.6-7.5
City	NO	NO Daily test	141 - 180	161 - 180	6.6-7.5
City	NO	NO Daily test	101 - 125	141 - 160	6.6-7.5
City	NO	NO Daily test	101 - 125	<140	6.6-7.5
City	NO	NO Daily test	101 - 125	<140	<6.5
City	NO	NO Daily test	<100	141 - 160	>9.6
City	NO	NO Daily test	101 - 125	<140	6.6-7.5
City	NO	NO Daily test	141 - 180	>180	8.6-9.5
City	YES	>3 times	101 - 125	161 - 180	<6.5
City	NO	NO Daily test	>180	>180	7.6-8.5
City	NO	NO Daily test	>180	>180	>9.6
Urban	NO	NO Daily test	<100	<140	6.6-7.5
Urban	NO	NO Daily test	>180	>180	7.6-8.5
City	NO	NO Daily test	101 - 125	>180	7.6-8.5
City	YES	Twice	101 - 125	141 - 160	6.6-7.5
City	YES	Once	>180	>180	>9.6
City	YES	Trice	141 - 180	>180	7.6-8.5
City	NO	NO Daily test	101 - 125	>180	8.6-9.5
City	NO	NO Daily test	<100	141 - 160	>9.6
City	NO	NO Daily test	101 - 125	161 - 180	7.6-8.5
City	NO	NO Daily test	<100	>180	>9.6
City	NO	NO Daily test	<100	<140	6.6-7.5
City	YES	Once	101 - 125	>180	6.6-7.5
City	NO	NO Daily test	126 - 140	>180	7.6-8.5

Duration of DM ()	Adherence of DM drugs	Overall Satis with diab care	other disease ?	cardiovascular
2	Always	Satisfied	NO	NO
4	Always	Satisfied	hypertension	Yes
	Often	Neutral	hypertension	Yes
8	Always	Satisfied	NO	NO
15	Always	Satisfied	spondylolisthes	NO
1	Always	Satisfied	NO	NO
14	Often	Satisfied	colon, spondylo	NO
19	Always	Neutral	cardio disease	Yes
6	Sometimes	Satisfied	NO	NO
15	Always	Satisfied	hypertension ,	Yes
3	Often	Satisfied	NO	NO
4	Always	Satisfied	hypertension	Yes
10	Often	Satisfied	hypertension	Yes
4	Always	Neutral	NO	NO
8	Always	Neutral	NO	NO
30	Always	Satisfied	NO	NO
10	Often	Satisfied	NO	NO
3	Always	Satisfied	NO	NO
8	Always	Satisfied	NO	NO
6	Sometimes	Satisfied	NO	NO
15	Often	Satisfied	colon, spondylo	NO
19	Always	Neutral	cardio disease	Yes
15	Sometimes	Satisfied	NO	NO
15	Always	Satisfied	hypertension ,	Yes
4	Often	Satisfied	NO	NO
4	Always	Satisfied	hypertension	Yes
10	Often	Satisfied	hypertension	Yes
8	Always	Satisfied	NO	NO
13	Always	Satisfied	spondylolisthes	NO
1	Always	Satisfied	NO	NO
14	Often	Satisfied	colon, spondylo	NO
17	Always	Neutral	cardio disease	Yes
	Always	Satisfied		NO
	Always	Satisfied		NO
	Often	Satisfied		NO
15	Always	Satisfied	hypertension	Yes
10	Always	Neutral	hypertension	Yes
25	Sometimes	Satisfied	hypertension	Yes
7	Often	Satisfied	hypertension	Yes
5	Always	Satisfied	NO	NO
20	Sometimes	Satisfied	NO	NO
13	Always	Satisfied		NO
4	Often	Neutral	NO	NO
1	Always	Neutral	NO	NO
9	Often	Satisfied	NO	NO
1	Always	Satisfied		NO
1	Always	Satisfied	NO	NO
1	Always	Satisfied	anemia	NO

2	Always	Satisfied	hypertension	Yes
15	Often	Satisfied	NO	NO
20	Always	Neutral	NO	NO
24	Always	Satisfied	NO	NO
11	Sometimes	Satisfied	NO	NO
4	Often	Satisfied	NO	NO
20	Always	Satisfied	NO	NO
2	Sometimes	Satisfied	NO	NO
3	Always	Neutral	NO	NO
15	Often	Neutral	hypertension	Yes
	Always	Satisfied		NO
18	Often	Satisfied	NO	NO
5	Always	Satisfied	NO	NO
	Always	Satisfied		NO
30	Always	Satisfied	hypertension,th	Yes
2	Always	Satisfied	hypertension ,	Yes
35	Often	Neutral	hypertension	Yes
2	Always	Satisfied	hypertension	Yes
1	Always	Satisfied		NO
4	Sometimes	Satisfied	hypertension	Yes
6	Often	Satisfied	hypertension	Yes
6	Always	Satisfied	NO	NO
2	Sometimes	Neutral	Hypertension a	Yes
1	Always	Neutral	NO	NO
	Often	Satisfied	hypertension, k	Yes
5	Always	Satisfied	Hypertension	Yes
5	Often	Satisfied	hypertension	Yes
2	Always	Satisfied	NO	NO
7	Always	Satisfied	Thyroid diseas	NO
5	Always	Satisfied	hypertension	Yes
15	Always	Neutral		NO
11	Often	Satisfied	NO	NO
	Always	Satisfied	hypertension	Yes
52	Always	Satisfied	hypertension	Yes
10	Sometimes	Satisfied	hypertension, k	Yes
56	Often	Satisfied	hypertension	Yes
18	Always	Neutral	NO	NO
18	Sometimes	Neutral	NO	NO
11	Always	Satisfied	hypertension	Yes
20	Often	Satisfied	NO	NO
20	Always	Satisfied	HF+hypertensi	Yes
15	Often	Satisfied	NO	NO
6	Always	Satisfied	NO	NO
15	Always	Satisfied	skin disease	NO
2	Often	Neutral	hypertension	Yes
5	Always	Satisfied	hypertension	Yes
15	Always	Satisfied	wheat allergy	NO
10	Always	Satisfied	NO	NO
10	Always	Satisfied	NO	NO

10	Often	Satisfied	NO	NO
10	Always	Neutral	NO	NO
10	Always	Neutral	NO	NO
11	Often	Satisfied	hypertension	Yes
	Always	Satisfied		NO
3	Always	Satisfied	hyperlipidemia	Yes
2	Always	Satisfied		NO
2	Always	Satisfied	NO	NO
50	Often	Satisfied	NO	NO
2	Always	Neutral	NO	NO
11	Always	Satisfied	NO	NO
10	Often	Satisfied	NO	NO
10	Always	Satisfied	NO	NO
10	Always	Satisfied	hypertension ,	Yes
10	Always	Satisfied	NO	NO
8	Always	Neutral	hyperlipidemia	Yes
17	Often	Neutral	kidney stones	NO
20	Always	Satisfied	Hypertension	Yes
19	Always	Satisfied	Atherosclerosi	Yes
49	Often	Satisfied	hypertension	Yes
17	Always	Satisfied	hypertension ,h	Yes
5	Always	Satisfied	NO	NO
17	Always	Satisfied	hypertension,h	Yes
17	Always	Neutral	hypertension,h	Yes
4	Often	Satisfied	NO	NO
2	Always	Satisfied	hypertension,a	Yes
15	Always	Satisfied	hypertension	Yes
1	Often	Satisfied	NO	NO
9	Always	Satisfied	hypertension	Yes
	Always	Neutral	NO	NO
7	Sometimes	Neutral		NO
10	Often	Satisfied	hypertension	Yes
15	Always	Satisfied	NO	NO
4	Sometimes	Satisfied	cyst in the brai	NO
15	Always	Satisfied	NO	NO
1	Often	Satisfied		NO
10	Always	Satisfied	NO	NO
10	Often	Neutral	NO	NO
10	Always	Satisfied	NO	NO
10	Always	Satisfied		NO
50	Often	Satisfied		NO
4	Always	Satisfied	hypertension	Yes
10	Always	Satisfied	NO	NO
30	Always	Neutral	hypertension	Yes
5	Always	Neutral	hypertension, a	Yes
17	Often	Satisfied	NO	NO
23	Always	Satisfied		NO
5	Always	Satisfied	NO	NO
	Often	Satisfied	NO	NO
25	Always	Satisfied	hypertension,h	Yes
1	Always	Satisfied	NO	NO
10	Always	Neutral	hypertension	Yes
	Always	Satisfied		NO
10	Often	Satisfied	NO	NO
15	Always	Satisfied	atherosclerosis	Yes
9	Always	Satisfied	NO	NO
10	Often	Satisfied	NO	NO
4	Always	Neutral	NO	NO

15	Always	Neutral	NO	NO
15	Always	Satisfied	hypertension	NO
1.5	Always	Satisfied	NO	Yes
1	Often	Satisfied	pituitary tumor	NO
10	Always	Satisfied	Hypertension a	Yes
22	Always	Satisfied	NO	NO
7	Often	Satisfied	NO	NO
8	Always	Neutral	NO	NO
15	Always	Satisfied	NO	NO
20	Always	Satisfied	NO	NO
	Always	Satisfied	hypertension	Yes
1	Often	Satisfied	NO	NO
14	Always	Satisfied	NO	NO
15	Always	Neutral	NO	NO
1	Often	Neutral	NO	NO
3	Always	Satisfied	NO	NO
11	Always	Satisfied	NO	NO
3	Sometimes	Satisfied	NO	NO
5	Often	Satisfied	NO	NO
10	Always	Satisfied	NO	NO
1	Sometimes	Neutral	NO	NO
	Always	Neutral	NO	NO
	Often	Satisfied	NO	NO
1	Always	Satisfied	NO	NO
12	Often	Satisfied	NO	NO
24	Always	Satisfied	Ceilac disease	Yes
27	Always	Satisfied	Coronary artery	Yes
20	Often	Satisfied	stomach ulcer	NO
1	Always	Neutral	NO	NO
30	Always	Satisfied	Hypertension	Yes
10	Sometimes	Satisfied	Hypothyrodism	NO
10	Often	Satisfied	Hypertension ,I	Yes
7	Always	Neutral	Hypertension	Yes
11	Sometimes	Neutral	Hypertension	Yes
5	Always	Satisfied	NO	NO
5	Often	Satisfied	Hypertension, I	Yes
1	Always	Satisfied	Hypertension	Yes
4	Often	Satisfied	Hypertension	Yes
15	Always	Satisfied	Hypertension	Yes
1	Always	Satisfied	NO	NO
10	Often	Neutral	NO	NO
15	Always	Satisfied	NO	NO
	Always	Satisfied	hypertension	Yes
10	Sometimes	Neutral	NO	NO
15	Often	Satisfied	hypertension	Yes
8	Always	Satisfied	NO	NO
1	Sometimes	Satisfied	NO	NO
20	Always	Satisfied		NO
8	Often	Satisfied	NO	NO
4	Always	Neutral	NO	NO
7	Often	Neutral	Sinusitis ,	NO
20	Always	Satisfied	Hypertension ,	Yes
8	Always	Satisfied	NO	NO
1	Often	Satisfied	NO	NO
10	Always	Satisfied	NO	NO
4	Often	Satisfied	NO	NO
15	Always	Satisfied	NO	NO

other disease	Worry enough foo	Decrease No./size	Decrease Quality	Hunger?cant affor	lost Wt no food
NO	Never	Often	Often	Never	Never
NO	Sometimes	Often	Rarely	Never	Never
NO	Often	Always	Always	Never	Never
Yes	Never	Often	Sometimes	Never	Never
Yes	Rarely	Sometimes	Often	Never	Never
NO	Never	Sometimes	Never	Never	Never
Yes	Sometimes	Sometimes	Sometimes	Never	Never
NO	Sometimes	Rarely	Never	Never	Never
NO	Never	Rarely	Rarely	Never	Never
Yes	Often	Always	Sometimes	Rarely	Sometimes
NO	Never	Sometimes	Never	Never	Never
NO	Never	Sometimes	Rarely	Never	Rarely
NO	Rarely	Often	Sometimes	Never	Never
NO	Often	Often	Never	Never	Often
NO	Sometimes	Often	Rarely	Never	Never
NO	Never	Sometimes	Never	Never	Never
NO	Rarely	Never	Sometimes	Never	Never
NO	Never	Sometimes	Never	Never	Never
NO	Never	Often	Rarely	Never	Never
NO	Sometimes	Rarely	Sometimes	Rarely	Never
Yes	Sometimes	Sometimes	Sometimes	Never	Never
NO	Sometimes	Rarely	Never	Never	Never
NO	Never	Rarely	Rarely	Never	Never
Yes	Often	Always	Sometimes	Rarely	Sometimes
NO	Never	Sometimes	Never	Never	Never
NO	Never	Sometimes	Rarely	Never	Rarely
NO	Rarely	Often	Sometimes	Never	Never
NO	Never	Often	Sometimes	Never	Never
Yes	Rarely	Sometimes	Often	Never	Never
NO	Never	Sometimes	Never	Never	Never
Yes	Sometimes	Sometimes	Sometimes	Never	Never
NO	Sometimes	Rarely	Never	Never	Never
NO	Never	Sometimes	Rarely	Never	Never
NO	Never	Often	Never	Never	Never
NO	Rarely	Often	Rarely	Rarely	Never
NO	Sometimes	Rarely	Sometimes	Rarely	Never
NO	Sometimes	Often	Sometimes	Rarely	Sometimes
NO	Never	Never	Never	Never	Never
NO	Often	Often	Never	Never	Often
NO	Sometimes	Sometimes	Never	Never	Never
NO	Rarely	Rarely	Sometimes	Rarely	Rarely
NO	Sometimes	Always	Sometimes	Often	Always
NO	Rarely	Sometimes	Rarely	Never	Never
NO	Rarely	Sometimes	Sometimes	Never	Never
NO	Rarely	Always	Often	Never	Never
NO	Never	Never	Rarely	Never	Never
NO	Sometimes	Sometimes	Always	Rarely	Rarely
Yes	Sometimes	Never	Never	Often	Always

NO	Rarely	Sometimes	Rarely	Never	Never
NO	Never	Always	Never	Never	Never
NO	Never	Always	Sometimes	Never	Never
NO	Rarely	Often	Rarely	Never	Often
NO	Never	Always	Never	Never	Never
NO	Often	Always	Sometimes	Never	Sometimes
NO	Rarely	Rarely	Sometimes	Rarely	Rarely
NO	Rarely	Always	Sometimes	Rarely	Never
NO	Never	Sometimes	Rarely	Rarely	Never
NO	Never	Often	Rarely	Never	Never
NO	Sometimes	Often	Rarely	Rarely	Never
NO	Sometimes	Rarely	Never	Never	Never
NO	Rarely	Sometimes	Never	Never	Rarely
NO	Often	Never	Never	Sometimes	Rarely
Yes	Never	Sometimes	Sometimes	Never	Never
Yes	Never	Often	Never	Never	Never
NO	Sometimes	Often	Often	Sometimes	Often
NO	Never	Never	Never	Never	Never
NO	Never	sometimes	Never	Never	Never
NO	Never	Never	Never	Never	Never
NO	Rarely	Often	Often	Sometimes	Rarely
NO	Never	Never	Never	Never	Never
Yes	Never	Sometimes	Never	Never	Never
NO	Sometimes	Often	Often	Sometimes	Often
Yes	Rarely	Never	Often	Never	Never
NO	Never	Often	Never	Never	Never
NO	Never	Often	Rarely	Never	Never
NO	Never	Sometimes	Never	Never	Never
Yes	Never	Never	Never	Never	Never
NO	Never	Sometimes	Rarely	Never	Never
NO	Never	Sometimes	Rarely	Never	Never
NO	Sometimes	Always	Sometimes	Never	Never
NO	Rarely	Sometimes	Never	Never	Never
NO	Sometimes	Sometimes	Rarely	Rarely	Never
Yes	Never	Always	Never	Never	Never
NO	Rarely	Sometimes	Rarely	Rarely	Never
NO	Always	Always	Sometimes	Never	Never
NO	Always	Always	Always	Never	Never
NO	Rarely	Sometimes	Rarely	Never	Never
NO	Never	Sometimes	Rarely	Sometimes	Rarely
NO	Never	Always	Often	Never	Never
NO	Never	Never	Never	Never	Never
NO	Never	Sometimes	Rarely	Never	Never
Yes	Never	Always	Often	Rarely	Always
NO	Sometimes	Always	Always	Sometimes	Often
NO	Never	Sometimes	Rarely	Never	Never
Yes	Sometimes	Rarely	Sometimes	Often	Never
NO	Rarely	Sometimes	Sometimes	Never	Never
NO	Rarely	Sometimes	Sometimes	Never	Never

NO	Rarely	Sometimes	Sometimes	Never	Never
NO	Rarely	Sometimes	Sometimes	Never	Never
NO	Rarely	Sometimes	Sometimes	Never	Never
NO	Never	Often	Sometimes	Never	Never
NO		Rarely			
NO	Often	Rarely	Rarely	Never	Never
NO	Never	Sometimes	Sometimes	Never	Never
NO	Sometimes	Often	Always	Sometimes	Often
NO	Often	Sometimes		Never	Never
NO	Sometimes	Often	Often	Never	Often
NO	Sometimes	Never	Never	Never	Never
NO	Never	Sometimes	Sometimes	Never	Never
NO	Never	Sometimes	Sometimes	Never	Never
NO	Never	Rarely	Never	Never	Never
NO	Never	Sometimes	Sometimes	Never	Never
NO	Never	Often	Never	Never	Never
Yes	Never	Always	Rarely	Rarely	Sometimes
NO	Rarely	Rarely	Sometimes	Never	Never
NO	Rarely	Often	Rarely	Never	Rarely
NO	Never	Sometimes	Never	Never	Never
Yes	Never	Never	Never	Never	Often
NO	Never	Sometimes	Never	Never	Never
Yes	Sometimes	Never	Sometimes	Never	Never
Yes	Never	Never	Sometimes	Never	Never
NO	Sometimes	Sometimes	Rarely	Never	Never
Yes	Never	never	Never	Never	Never
NO	Never	Never	Rarely	Never	Never
NO	Sometimes	Sometimes	Often	Rarely	Sometimes
NO	Never	Often	Never	Never	Never
NO	Sometimes	Sometimes	Often	Rarely	Sometimes
NO	Never	Sometimes	Rarely	Never	Sometimes
NO	Rarely	Sometimes	Sometimes	Rarely	Rarely
NO	Rarely	Often	Often	Rarely	Often
Yes	Rarely	Often	Never	Never	Never
NO	Never	Never	Never	Never	Never
NO	Never	Always	Often	Never	Never
NO	Never	Sometimes	Sometimes	Never	Never
NO	Never	Sometimes	Sometimes	Never	Never
NO	Never	Sometimes	Sometimes	Never	Never
NO	Rarely	Sometimes	Never	Never	Rarely
NO	Rarely	Often	Never	Rarely	Never
NO	Never	Often	Often	Never	Often
NO	Sometimes	Often	Sometimes	Never	Sometimes
NO	Sometimes	Rarely	Sometimes	Never	Never
NO	Sometimes	Often	Often	Sometimes	Always
NO	Never	Sometimes	Never	Rarely	Never
NO	Always	Never	Never	Never	Often
NO	Sometimes	Often	Sometimes	Sometimes	Sometimes
NO	Never	Never	Never	Never	Never
NO	Rarely	Sometimes	Sometimes	Never	Never
NO	Often	Always	Often	Rarely	Sometimes
NO	Often	Sometimes	Always	Sometimes	Sometimes
NO	Never	Sometimes	Rarely	Never	Never
NO	Sometimes	Often	Sometimes	Often	Sometimes
NO	Sometimes	Sometimes	Often	Never	Never
NO	Never	Sometimes	Sometimes	Never	Never
NO	Never	Sometimes	Sometimes	Never	Rarely
NO	Never	Sometimes	Never	Never	Never

NO	Often	Often	Never	Never	Never
NO	Rarely	Sometimes	Sometimes	Never	Never
NO	Rarely	Sometimes	Never	Never	Never
Yes	Never	Sometimes	Rarely	Never	Never
NO	Never	Rarely	Never	Never	Never
NO	Never	Sometimes	Rarely	Never	Never
NO	Rarely	Rarely	Never	Never	Never
NO	Never	Rarely	Never	Never	Never
NO	Never	Never	Never	Never	Never
NO	Often	Sometimes	Rarely	Sometimes	Often
NO	Sometimes	Sometimes	Rarely	Rarely	Sometimes
NO	Never	Often	Often	Often	Never
NO	Never	Sometimes	Never	Never	Never
NO	Sometimes	Rarely	Never	Never	Never
NO	Never	Always	Never	Never	Never
NO	Sometimes	Sometimes	Sometimes	Sometimes	Always
NO	Never	Often	Never	Never	Never
NO	Sometimes	Often	Often	Rarely	Rarely
NO	Never	Sometimes	Rarely	Rarely	Never
NO	Sometimes	Often	Often	Rarely	Rarely
NO	Sometimes	Often	Rarely	Never	Rarely
NO	Sometimes	Often	Often	Rarely	Rarely
NO	Sometimes	Rarely	Never	Never	Never
NO	Rarely	Rarely	Rarely	Rarely	Rarely
NO	Rarely	Rarely	Rarely	Rarely	Rarely
NO	Rarely	Rarely	Rarely	Rarely	Rarely
NO	Never	Never	Never	Never	Never
Yes	Always	Always	Always	Always	Always
NO	Rarely	Sometimes	Rarely	Rarely	Rarely
NO	Often	Often	Often	Often	Often
Yes	Rarely	Rarely	Rarely	Rarely	Rarely
Yes	Always	Always	Always	Always	Sometimes
NO	Often	Always	Always	Often	Sometimes
NO	Never	Never	Never	Never	Never
NO	Sometimes	Never	Never	Never	Never
Yes	Never	Often	Rarely	Never	Never
NO	Never	Sometimes	Rarely	Never	Never
NO	Never	Sometimes	Never	Never	Never
NO	Never	Never	Never	Never	Never
NO	Always	Sometimes	Never	Never	Never
NO	Rarely	Often	Rarely	Rarely	Never
NO	Never	Never	Never	Never	Never
NO	Always	Always	Always	Often	Always
NO	Never	Rarely	Never	Never	Never
NO	Always	Always	Always	Often	Never
NO	Often	Often	Sometimes	Rarely	Never
NO	Always	Always	Always	Sometimes	Rarely
NO	Often	Always	Sometimes	Often	Always
NO	Never	Never	Never	Never	Never
NO	Rarely	Always	Always	Rarely	Rarely
Yes	Never	Never	Sometimes	Never	Never
NO	Never	Rarely	Rarely	Never	Never
NO	Never	Sometimes	Never	Never	Never
NO	Always	Always	Sometimes	Never	Never
NO	Always	Rarely	Often	Always	Always
NO	Rarely	Rarely	Rarely	Rarely	Rarely
NO	Never	Never	Never	Never	Never

hunger ?cant slee	Evacuated before?	ForceClosed?	Homeless/shelter?	Reason to MOVE	if other reseons pls typ
Never	NO	NO	NO		
Always	YES	YES	YES	Rent increases	
Never	NO	NO	NO		
Never	NO	NO	NO		
Rarely	YES	NO	NO	Other reasons	husband died
Never	YES	NO	NO	s or maintenance issues	
Never	NO	NO	NO		
Never	NO	NO	NO		
Sometimes	NO	NO	NO	Other reasons	doesnot like the city
Sometimes	NO	NO	NO		
Never	NO	NO	NO		
Sometimes	NO	NO	NO	Other reasons	move to better house
Never	NO	NO	NO	Rent increases	
Never	NO	NO	NO		
Rarely	NO	NO	NO	s or maintenance issues	
Never	NO	NO	NO		
Rarely	NO	NO	NO	Rent increases	
Never	NO	NO	NO		
Never	NO	NO	NO	Other reasons	move to better house
Sometimes	NO	NO	NO		
Never	NO	NO	NO		
Never	NO	NO	NO		
Sometimes	NO	NO	NO	andlord harassment	
Sometimes	NO	NO	NO		
Never	NO	NO	NO		
Sometimes	NO	NO	NO	Rent increases	
Never	NO	NO	NO	Rent increases	
Never	NO	NO	NO		
Rarely	YES	NO	NO	Rent increases	
Never	YES	NO	NO	Rent increases	
Never	NO	NO	NO		
Never	NO	NO	NO		
Never	NO		NO		
Sometimes	NO	NO	NO		
Rarely	NO	YES	YES		
Never	NO	NO	NO	s or maintenance issues	
Always	NO	NO	NO	s or maintenance issues	
Never	NO	NO	NO		
Always	NO	NO	NO	NO	
Never	YES	YES	NO	Rent increases	
Never	NO	NO	YES	Other reasons	
Often	NO	YES	YES	Rent increases	
Sometimes	NO	NO	NO		
Rarely	NO	NO	NO		
Rarely	NO	NO	NO	s or maintenance issues	
Never	NO	NO	NO	Other reasons	
Sometimes	NO	NO	NO	Other reasons	
Never	NO	NO	NO	s or maintenance issues	

Never	NO	NO	NO	NO	
Never	NO	NO	NO	NO	
Never	YES	NO	YES	Other reasons	marital problems
Never	NO	NO	YES	Other reasons	maritof problems
Rarely	NO	NO	NO		
Often	NO	NO	NO	Other reasons	
Never	NO	NO	YES	Other reasons	
Always	NO	NO		Other reasons	
Always	NO	NO	NO	s or maintenance issues	
Often	NO	NO	NO	s or maintenance issues	
Never	NO	NO	NO	Rent increases	
Never	NO	NO	NO		
Sometimes	NO	NO	YES	Other reasons	
Sometimes	YES	NO	NO	Other reasons	
Never	NO	NO	NO	s or maintenance issues	
Sometimes	NO	NO	NO		
Sometimes	YES	NO	YES	Rent increases	
Never	NO	NO	NO		
Never	NO	NO	NO		
Never	NO	NO	NO		
Often	NO	NO	NO	Other reasons	
Never	NO	NO	NO	s or maintenance issues	
Never	NO	NO	NO	other reasons	the house is small
Often	NO	NO	NO	Other reasons	
Often	NO	NO	NO		
Rarely	YES	YES	YES	Other reasons	financial problem
Rarely	YES	YES	YES	Rent increases	
Never	NO	NO	NO		
Never	NO	NO	NO		
Rarely	YES	YES	YES	Rent increases	
Sometimes	NO	NO	NO	Other reasons	
Never	NO	NO	NO		
Never	NO	NO	NO	s or maintenance issues	
Rarely	YES	NO	YES	Rent increases	
Rarely	NO	NO	NO	no	
Rarely	YES	NO	NO	Rent increases	
Often	NO	NO	NO		
Often	NO	NO	NO		
Never	NO	NO	NO	Rent increases	
Often	NO	NO	NO	s or maintenance issues	
Never	NO	NO	NO	s or maintenance issues	
Never	NO	NO	NO	s or maintenance issues	
Rarely	NO	NO	NO	Other reasons	
Rarely	NO	NO	NO		
Rarely	NO	NO	NO	Other reasons	
Never	NO	NO	NO	Other reasons	
Never	NO	NO	NO	Rent increases	
Rarely	NO	NO	NO	Other reasons	financial problem
Rarely	NO	NO	NO	Other reasons	financial problem

Rarely	NO	NO	NO	Other reasons	financial problem
Rarely	NO	NO	NO	Other reasons	financial problem
Rarely	NO	NO	NO	Other reasons	financial problem
Never	NO	NO	NO	no	
Often	NO	NO	NO		
Never	NO	NO	NO	Other reasons	
Often	NO	NO	NO		
Sometimes	YES	NO	YES	andlord harassment	
Often	NO	NO	NO		
Rarely	NO	NO	NO		
Rarely	NO	NO	NO	Other reasons	financial problem
Rarely	NO	NO	NO	Other reasons	financial problem
Never	NO	NO	NO	no	
Rarely	NO	NO	NO	Other reasons	financial problem
Rarely	NO	NO	NO	andlord harassment	
Often	NO	NO	NO	no	
Never	NO	NO	NO	Rent increases	
Rarely	NO	NO	NO	s or maintenance issues	
Rarely	NO	NO	NO	Other reasons	
Always	NO	NO	NO	Other reasons	
Never	NO	NO	NO	Other reasons	
Always	NO	NO	NO	Other reasons	
Always	NO	NO	NO	Other reasons	
Never	NO	NO	NO	s or maintenance issues	
Never	NO	NO	NO		
Never	NO	NO	NO	s or maintenance issues	
Rarely	NO	NO	NO		
Never	NO	NO	NO		
Rarely	NO	NO	NO		
Often	NO	NO	NO		
Sometimes	NO	NO	NO		
Sometimes	NO	NO	NO	Rent increases	
Never	NO	NO	NO	s or maintenance issues	
Rarely	NO	NO	NO	s or maintenance issues	
Never	NO	NO	NO	s or maintenance issues	
Rarely	NO	NO	NO	Other reasons	financial problem
Rarely	NO	NO	NO	Other reasons	financial problem
Rarely	NO	NO	NO	Other reasons	financial problem
Never	NO	YES	NO	Rent increases	
Rarely	NO	NO	YES		
Sometimes	NO	NO	NO	s or maintenance issues	
Often	NO	NO	YES	s or maintenance issues	
Sometimes	NO	NO	NO		
Sometimes	NO	NO	NO	Other reasons	
Rarely	NO	NO	YES	Rent increases	
Always	NO	NO	NO	Other reasons	for study
Sometimes	NO	NO	NO	s or maintenance issues	
Rarely	NO	NO	NO	s or maintenance issues	
Never	NO	NO	NO	no	
Rarely	NO	NO	NO	s or maintenance issues	
Never	NO	NO	NO	Other reasons	financial problem
Never	NO	NO	NO		
Often	NO	NO	NO	andlord harassment	
Sometimes	NO	NO	YES	no	
Rarely	NO	NO	NO	s or maintenance issues	
Never	YES	NO	NO		
Never	NO	NO	NO		

Often	NO	NO	NO		
Never	NO	NO	NO	s or maintenance issues	
Never	NO	NO	NO		
Never	NO	NO	NO	Other reasons	
Sometimes	NO	NO	NO		
Never	NO	NO	NO		
Never	NO	NO	NO	s or maintenance issues	
Never	NO	NO	NO	Other reasons	
Never	NO	NO	NO	Other reasons	
Often	NO	NO	NO	Rent increases	
Sometimes	NO	NO	NO	s or maintenance issues	
Never	YES	YES	NO	Rent increases	
Never	NO	NO	NO		
Never	NO	NO	NO		
Never	NO	NO	NO		
Always	NO	NO	NO	s or maintenance issues	
Rarely	NO	NO	NO		
Rarely	NO	NO	NO	Rent increases	
Rarely	NO	NO	NO		
Rarely	NO	NO	NO	s or maintenance issues	
Sometimes	YES	YES	YES	andlord harassment	
Rarely	NO	NO	NO	s or maintenance issues	
Sometimes	NO	NO	NO		
Rarely	NO	NO	NO		
Often	NO	NO	NO		
Often	NO	NO	NO		
Never	NO	NO	NO		
Always	NO	NO	NO		
Sometimes	NO	NO	NO		
Sometimes	NO	NO	NO		
Always	NO	NO	NO		
Always	NO	NO	NO		
Always	YES	YES	NO	Rent increases	
Often	NO	NO	NO		
Never	NO	NO	NO		
Rarely	YES	NO	NO	andlord harassment	
Never	YES	NO	NO	Other reasons	forced displacement
Never	YES	NO	NO	andlord harassment	
Never	NO	NO	NO	Rent increases	
Never	NO	NO	NO		
Sometimes	NO	NO	YES	s or maintenance issues	
Never	YES	NO	NO	s or maintenance issues	
Rarely	YES	NO	YES	Other reasons	forced displacement
Never	YES	NO	YES	andlord harassment	
Always	NO	NO	NO		
Sometimes	NO	NO	NO		
Sometimes	NO	NO	NO		
Often	NO	NO	NO	s or maintenance issues	
Never	YES	NO	NO	Other reasons	
Rarely	NO	NO	NO	s or maintenance issues	
Rarely	NO	NO	NO	s or maintenance issues	
Never	YES	NO	NO	andlord harassment	
Never	NO	NO	NO		
Sometimes	NO	NO	NO		
Always	NO	NO	NO	Other reasons	
Rarely	NO	NO	NO		
Never	NO	NO	YES	Other reasons	sectarianism

Worry aford rent/mortage	How often feel safe in home	have people emotional suppor	Have people for help'
Often	Always	Always	Always
Always	Sometimes	Never	Always
Never	Often	Often	Always
Never	Sometimes	Never	Never
Sometimes	Sometimes	Sometimes	Sometimes
Sometimes	Always	Often	Sometimes
Never	Always	Always	Always
Sometimes	Always	Often	Often
Never	Always	Always	Always
Never	Often	Often	Often
Never	Always	Often	Often
Never	Always	Often	Always
Sometimes	Often	Often	Often
Never	Always	Always	Always
Never	Always	Sometimes	Always
Never	Always	Always	Always
Often	Often	Always	Sometimes
Never	Always	Often	Often
Never	Always	Always	Often
Never	Always	Never	Never
Never	Always	Always	Always
Sometimes	Always	Often	Often
Never	Always	Always	Always
Never	Often	Often	Often
Never	Always	Often	Often
Never	Always	Often	Always
Sometimes	Often	Often	Often
Never	Sometimes	Never	Never
Sometimes	Sometimes	Sometimes	Sometimes
Sometimes	Always	Often	Sometimes
Never	Always	Always	Always
Sometimes	Always	Often	Often
Always	Always	Sometimes	Always
Always	Always	Always	Always
Often	Sometimes	Often	Sometimes
Always	Rarely	Never	Often
Rarely	Sometimes	Always	Always
Always	Always	Always	Always
Always	Always	Always	Rarely
Always	Sometimes	Often	Often
Always	Sometimes	Always	Often
Always	Sometimes	Often	Sometimes
Always	Often	Often	Often
Always	Often	Often	Sometimes
Often	Always	Often	Sometimes
Always	Always	Always	Always
Always	Sometimes	Often	Sometimes
Rarely	Sometimes	Rarely	Rarely

Always	Always	Always	Always
Always	Always	Always	Always
Always	Often	Often	Often
Always	Often	Often	Often
Always	Always	Always	Always
Often	Rarely	Never	Never
Always	Sometimes	Always	Often
Always	Always	Always	Always
Always	Always	Always	Always
Always	Always	Often	Sometimes
Always	Often	Often	Sometimes
Often	Sometimes	Often	Often
Often	Sometimes	Rarely	Rarely
Often	Always	Sometimes	Often
Always	Sometimes	Always	Always
Always	Never	Sometimes	Sometimes
Sometimes	Often	Often	Sometimes
Always	Always	Always	Always
Always	Always	Always	Always
Always	Always	Always	Always
Always	Always	Always	Always
Often	Sometimes	Sometimes	Sometimes
Always	Always	Always	Always
Often	Sometimes	Sometimes	Sometimes
Always	Always	Always	Always
Often	Always	Often	Rarely
Sometimes	Often	Often	Sometimes
Always	Often	Often	Sometimes
Always	Always	Always	Often
Often	Often	Often	Sometimes
Always	Always	Always	Always
Always	Always	Always	Always
Often	Always	Always	Sometimes
Often	Always	Often	Rarely
Always	Often	Sometimes	Sometimes
Often	Sometimes	Sometimes	Rarely
Always	Always	Sometimes	Often
Always	Often	Often	Sometimes
Always	Always	Often	Always
Always	Often	Often	Often
Always	Always	Always	Never
Always	Always	Always	Always
Always	Always	Sometimes	Rarely
Always	Often	Always	Always
Always	Often	Often	Sometimes
Often	Often	Always	Always
Never	Always	Often	Rarely
Often	Always	Always	Always
Often	Always	Always	Always

Often	Always	Always	Always
Often	Always	Always	Always
Often	Always	Always	Always
Always	Rarely	Sometimes	Rarely
	Often	Always	Often
Always	Always	Always	Rarely
Always	Often	Often	Sometimes
Sometimes	Always	Often	Often
Always	Always	Always	Always
Often	Often	Often	Often
Always	Often	Always	Often
Often	Always	Always	Always
Often	Always	Always	Always
Always	Always	Often	Always
Often	Always	Always	Always
Often	Always	Always	Often
Often	Sometimes	Sometimes	
Always	Always	Often	Always
Often	Always	Always	Often
Always	Often	Often	Often
Always	Always	Always	Always
Always	Always	Sometimes	Often
Always	Always	Always	Always
Always	Always	Always	Always
Always	Always	Often	Often
Often	Always	Always	Always
Always	Often	Often	Often
Often	Often	Always	Always
Always	Often	Always	Always
Often	Often	Always	Always
Always	Always	Always	Always
Often	Often	Sometimes	Rarely
Always	Always	Always	Often
Often	Sometimes	Often	Sometimes
Always	Often	Always	Always
Always	Often	Always	Often
Often	Always	Always	Always
Often	Always	Always	Always
Often	Always	Always	Always
Always	Often	Always	Always
Always	Often	Always	Always
Sometimes	Often	Always	Always
Rarely	Often	Always	Always
Always	Always	Always	Always
Always	Always	Always	Always
Often	Always	Always	Always
Often	Always	Always	Always
Always	Rarely	Rarely	Sometimes
Always	Often	Always	Always
Always	Sometimes	Always	Never
Always	Always	Always	Often
Often	Often	Often	Always
Always	Always	Always	Always
Sometimes	Often	Sometimes	Often
Always	Always	Always	Always
Always	Sometimes	Never	Rarely
Always	Often	Often	Sometimes
Always	Always	Always	Always

Always	Always	Often	Often
Always	Never	Sometimes	Sometimes
Always	Always	Sometimes	Always
Always	Often	Sometimes	Sometimes
Always	Always	Always	Always
Always	Always	Always	Always
Often	Always	Always	Sometimes
Always	Sometimes	Often	Always
Often	Always	Always	Often
Always	Often	Often	Always
Often	Always	Sometimes	Often
Always	Always	Always	Always
Always	Sometimes	Sometimes	Sometimes
Always	Always	Often	Often
Always	Always	Always	Always
Always	Often	Often	Often
Always	Often	Sometimes	Sometimes
Always	Always	Always	Always
Always	Often	Always	Always
Always	Often	Often	Often
Always	Sometimes	Sometimes	Often
Always	Always	Always	Always
Always	Sometimes	Often	Often
Always	Often	Often	Often
Sometimes	Often	Often	Often
Always	Often	Often	Always
Always	Rarely	Sometimes	Often
Sometimes	Often	Often	Never
Always	Always	Always	Sometimes
Often	Often	Often	Sometimes
Often	Often	Often	Often
Rarely	Often	Often	Often
Rarely	Always	Always	Often
Always	Often	Often	Always
Always	Always	Always	Always
Always	Sometimes	Sometimes	Rarely
Always	Often	Always	Always
Sometimes	Often	Always	Sometimes
Often	Often	Often	Often
Rarely	Sometimes	Sometimes	Sometimes
Always	Always	Always	Always
Always	Always	Always	Always
Always	Sometimes	Never	Never
Always	Sometimes	Sometimes	Always
Always	Often	Always	Always
Always	Always	Always	Often
Always	Often	Often	Often
Sometimes	Sometimes	Often	Often
Often	Often	Often	Sometimes
Always	Always	Always	Always
Often	Sometimes	Sometimes	Sometimes
Often	Rarely	Always	Rarely
Always	Always	Always	Always
Always	Always	Often	Always
Always	Often	Often	Never
Often	Often	Often	Often
Often	Sometimes	Sometimes	Often

Have people for scretes	Have people have fun with	feel belong to community	feel isolated/lonely?
Always	Always	Rarely	Always
Always	Often	Sometimes	Always
Always	Always	Always	Never
Never	Never	Always	Never
Sometimes	Rarely	Sometimes	Often
Often	Often	Always	Never
Always	Always	Always	Sometimes
Often	Often	Always	Never
Always	Always	Always	Never
Often	Sometimes	Sometimes	Sometimes
Often	Sometimes	Sometimes	Sometimes
Always	Always	Sometimes	Sometimes
Often	Often	Often	Rarely
Always	Always	Always	Never
Always	Often	Rarely	Always
Always	Sometimes	Sometimes	Always
Often	Sometimes	Sometimes	Rarely
Often	Sometimes	Sometimes	Never
Often	Sometimes	Always	Always
Never	Sometimes	Never	Never
Always	Always	Always	Sometimes
Often	Often	Always	Never
Always	Always	Always	Never
Often	Sometimes	Sometimes	Sometimes
Often	Sometimes	Sometimes	Sometimes
Always	Always	Sometimes	Sometimes
Often	Often	Often	Rarely
Never	Never	Always	Never
Sometimes	Rarely	Sometimes	Often
Often	Often	Always	Never
Always	Always	Always	Sometimes
Often	Often	Always	Never
Always	Always	Rarely	Often
Always	Often	Rarely	Often
Sometimes	Sometimes	Rarely	Often
Always	Always	Never	Often
Always	Always	Sometimes	Always
Often	Always	Rarely	Often
Rarely	Always	Sometimes	Rarely
Often	Always	Always	Often
Always	Rarely	Sometimes	Always
Rarely	Never	Rarely	Rarely
Often	Often	Rarely	Often
Sometimes	Rarely	Rarely	Sometimes
Often	Always	Often	Often
Always	Always	Never	Always
Often	Sometimes	Always	Often
Never	Never	Always	Never

Always	Always	Never	Never
Always	Always	Never	Never
Often	Often	Sometimes	Never
Often	Sometimes	Rarely	Never
Always	Always	Sometimes	Always
Sometimes	Often	Always	Sometimes
Always	Rarely	Sometimes	Always
Always	Sometimes	Sometimes	Always
Always	Always	Often	Always
Sometimes	Always	Sometimes	Sometimes
Often	Often	Sometimes	Often
Always	Always	Never	Always
Often	Rarely	Often	Often
Always	Sometimes	Never	Always
Always	Always	Rarely	Always
Always	Sometimes	Never	Always
Sometimes	Sometimes	Sometimes	Sometimes
Always	Always	Never	Always
Always	Often	Sometimes	Always
Always	Always	Never	Always
Always	Often	Rarely	Always
Sometimes	Often	Rarely	Sometimes
Often	Always	Rarely	Often
Sometimes	Sometimes	Sometimes	Sometimes
Always	Always	Sometimes	Always
Sometimes	Often	Sometimes	Sometimes
Sometimes	Often	Sometimes	Sometimes
Often	Sometimes	Rarely	Often
Sometimes	Sometimes	Sometimes	Sometimes
Often	Sometimes	Rarely	Often
Always	Often	Rarely	Always
Always	Always	Never	Always
Often	Always	Rarely	Often
Often	Rarely	Sometimes	Often
Often	Often	Rarely	Often
Often	Sometimes	Sometimes	Often
Always	Often	Never	Always
Sometimes	Often	Never	Sometimes
Often	Sometimes	Sometimes	Sometimes
Often	Often	Never	Sometimes
Often	Rarely	Always	Sometimes
Always	Sometimes	Sometimes	Always
Sometimes	Rarely	Sometimes	Sometimes
Often	Often	Sometimes	Often
Never	Always	Always	Never
Always	Often	Rarely	Always
Never	Rarely	Rarely	Never
Always	Sometimes	Sometimes	Always
Always	Sometimes	Sometimes	Always

Always	Sometimes	Sometimes	Always
Always	Sometimes	Sometimes	Always
Always	Sometimes	Sometimes	Always
Sometimes	Never	Always	Sometimes
Always	Sometimes	Rarely	Always
Always	Always	Never	Always
Sometimes	Often	Sometimes	Sometimes
Often	Always	Sometimes	Often
Always	Always		Always
Sometimes	Often	Sometimes	Sometimes
Always	Often	Never	Always
Always	Sometimes	Sometimes	Always
Always	Sometimes	Sometimes	Always
Always	Always	Never	Always
Always	Sometimes	Sometimes	Always
Often	Sometimes	Sometimes	Often
Often	Always	Often	Often
Often	Often	Sometimes	Often
Always	Often	Rarely	Often
Often	Sometimes	Never	Often
Always	Always	Never	Always
Sometimes	Sometimes	Rarely	Sometimes
Always	Always	Never	Sometimes
Always	Always	Never	Sometimes
Often	Always	Rarely	Sometimes
Always	Always	Never	Sometimes
Often	Always	Sometimes	Often
Always	Always	Never	Always
Sometimes	Sometimes	Sometimes	Sometimes
Always	Always	Never	Always
Always	Often	Often	Always
Sometimes	Often	Often	Sometimes
Always	Always	Often	Always
Often	Sometimes	Sometimes	Often
Often	Sometimes	Rarely	Often
Always	Always	Rarely	Always
Always	Sometimes	Sometimes	Always
Always	Sometimes	Sometimes	Always
Always	Sometimes	Often	Always
Always	Sometimes	Sometimes	Always
Always	Always	Rarely	Always
Always	Always	Never	Always
Sometimes	Sometimes	Always	Sometimes
Often	Always	Sometimes	Often
Always	Always	Rarely	Always
Always	Always	Rarely	Always
Always	Always	Sometimes	Always
Sometimes	Never	Always	Sometimes
Often	Often	Sometimes	Often
Always	Rarely	Sometimes	Always
Sometimes	Often	Rarely	Sometimes
Often	Often	Never	Often
Always	Often	Often	Always
Sometimes	Always	Always	Sometimes
Always	Always	Sometimes	Always
Rarely	Never	Always	Rarely
Often	Rarely	Sometimes	Often
Always	Often	Sometimes	Always

Often	Sometimes	Never	Often
Always	Sometimes	Sometimes	Always
Often	Often	Never	Often
Sometimes	Often	Rarely	Sometimes
Always	Sometimes	Sometimes	Always
Always	Always	Never	Always
Always	Rarely	Often	Always
Always	Often	Rarely	Always
Always	Often	Sometimes	Always
Rarely	Sometimes	Often	Rarely
Sometimes	Rarely	Often	Sometimes
Always	Always	Always	Always
Often	Often	Rarely	Often
Often	Often	Never	Often
Always	Often	Never	Always
Always	Always	Rarely	Always
Always	Always	Rarely	Always
Always	Always	Never	Always
Always	Always	Sometimes	Always
Often	Often	Sometimes	Often
Often	Rarely	Often	Often
Often	Sometimes	Sometimes	Often
Sometimes	Rarely	Always	Sometimes
Always	Always	Never	Always
Often	Sometimes	Sometimes	Often
Often	Always	Never	Often
Often	Never	Sometimes	Often
Never	Rarely	Often	Never
Sometimes	Sometimes	Never	Sometimes
Sometimes	Sometimes	Sometimes	Sometimes
Often	Often	Never	Often
	Sometimes	Always	
Often	Never	Rarely	Often
Always	Always	Never	Always
Always	Always	Never	Always
Sometimes	Always	Sometimes	Sometimes
Often	Often	Never	Often
Sometimes	Sometimes	Sometimes	Sometimes
Often	Always	Never	Often
Rarely	Never	Always	Rarely
Always	Always	Sometimes	Always
Always	Rarely	Rarely	Always
Sometimes	Sometimes	Always	Sometimes
Always	Sometimes	Rarely	Always
Always	Always	Never	Always
Often	Often	Never	Often
Often	Often	Rarely	Often
Often	Rarely	Sometimes	Often
Sometimes	Sometimes	Often	Sometimes
Always	Always	Sometimes	Always
Often	Always	Rarely	Often
Always	Always	Often	Always
Always	Always	Never	Always
Always	Always	Never	Always
Often	Often	Rarely	Often
Often	Often	Sometimes	Often
Often	Sometimes	Sometimes	Often

sumsupport
